# Gut and Intraplaque Microbiota in Atherosclerosis: Mechanistic Insights and Emerging Therapeutic Targets

**DOI:** 10.3390/microorganisms14102333

**Published:** 2026-10-13

**Authors:** Qijian Zhao, Zhichao Lai, Yuyao Feng, Jiang Shao, Kang Li, Shi Huang, Bao Liu

**Affiliations:** 1 Department of Vascular Surgery, Peking Union Medical College Hospital, Peking Union Medical College and Chinese Academy of Medical Sciences, Beijing 100730, China; zhaoqj0219@126.com (Q.Z.); laizhichao@pumch.cn (Z.L.); yuyao.feng@student.pumc.edu.cn (Y.F.); esan2002@vip.sina.com (J.S.); likang9010@163.com (K.L.); 2 Faculty of Dentistry, University of Hong Kong, Hong Kong SAR, China

**Keywords:** atherosclerosis, gut microbiota, intraplaque microbiota, metabolites, cardiovascular disease, therapeutic strategies

## Abstract

Atherosclerosis remains a major cause of cardiovascular morbidity and mortality. Growing evidence suggests that microbiota–host interactions contribute to atherosclerosis through systemic and local mechanisms. This review summarizes current evidence on gut microbiota, microbial metabolites, and plaque-associated microbial signals, and discusses their potential therapeutic implications. Gut dysbiosis may contribute to atherosclerosis through multiple metabolite-dependent, immune, vascular, and metabolic pathways. Recent multi-omics and experimental studies further indicate that microbiota influence vascular biology beyond compositional changes alone. In parallel, plaque-associated microbial signals, potentially originating from the oral cavity or gut, may contribute to local inflammation, oxidative stress, necrotic core expansion, and plaque vulnerability. We further discuss a putative gut–oral–intraplaque axis, a potential local microbial metabolite microenvironment within plaques, and possible cross-kingdom interactions between gut fungi and bacteria. Together, these findings support an integrated framework in which microbiota may shape atherosclerosis through interconnected systemic and local pathways, with potential implications for biomarker development and microbiota-targeted therapies.

## 1. Introduction

Atherosclerosis (AS) is the principal pathological basis of cardiovascular disease and ischemic stroke, characterized by chronic inflammation and metabolic dysregulation [1]. Although traditional risk factors such as hyperlipidemia, hypertension, and diabetes have been well established in AS development, adequate control of these factors does not fully eliminate the residual risk, suggesting that additional mechanisms remain insufficiently explained [2].

In recent years, the gut microbiota—the largest microbial ecosystem in the human body—has emerged as a new focus in cardiovascular research. By regulating lipid metabolism, immune homeostasis, and inflammatory responses, the gut microbiota exerts profound effects on host cardiovascular health [3]. Accumulating evidence links gut microbial dysbiosis and altered microbial metabolism to the development and progression of AS [4,5]. More intriguingly, some studies have detected bacterial DNA and other microbial signals within atherosclerotic plaques, highlighting the complexity of host–microbe interactions and raising the possibility that microbial components may be present within the arterial wall [6,7]. Such plaque-associated microbial signals may interact with the local microenvironment and potentially contribute to AS progression.

Although several reviews have summarized the relationship between the gut microbiota and atherosclerosis, this review extends beyond a primarily gut-centered perspective by integrating gut-derived metabolites, mechanistic evidence, and plaque-associated microbial signals within a systemic–local framework. Within this framework, we further consider the potential connections among gut dysbiosis, oral microorganisms, and plaque-associated microbial signals, while clearly distinguishing established evidence from proposed mechanisms. In particular, we critically evaluate the evidence for intraplaque microorganisms according to different microbial detection approaches and discuss potential sources of heterogeneity across intraplaque microbiome studies. In addition, incorporating recent studies, we categorize mechanistic evidence across human, animal, and cell-based research and provide a more detailed discussion of methodological limitations, confounding factors, and the translational evidence for microbiota-targeted therapies. Collectively, these perspectives aim to provide a more comprehensive and evidence-based view of microbiome involvement in atherosclerosis.

## 2. Pathogenic Mechanisms Linking Gut Microbial Dysbiosis to Atherosclerosis

**Figure 1:**
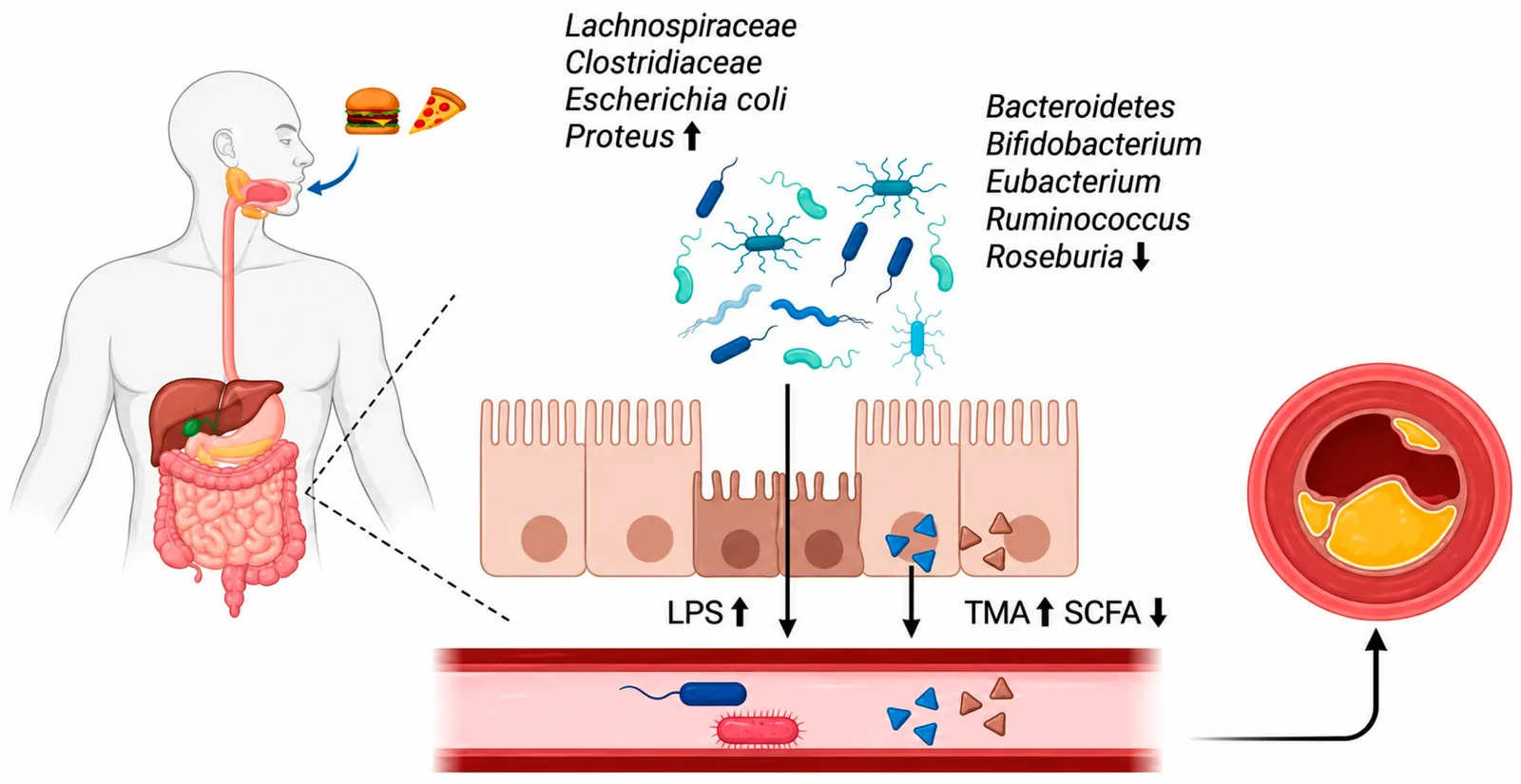
Gut microbial dysbiosis and metabolite-mediated mechanisms in atherosclerosis. High-fat dietary intake alters gut microbiota composition, characterized by an increase in potentially pathogenic taxa (e.g., *Lachnospiraceae, Clostridiaceae*, *Escherichia coli*, *Proteus*) and a reduction in commensal taxa associated with SCFA production and metabolic homeostasis (e.g., *Bacteroidetes*, *Bifidobacterium*, *Eubacterium*, *Ruminococcus*, *Roseburia*). Dysbiosis disrupts intestinal barrier integrity and modifies microbial metabolism, leading to elevated levels of lipopolysaccharides (LPS) and trimethylamine (TMA), together with reduced short-chain fatty acids (SCFAs). These changes promote systemic inflammation, endothelial dysfunction, and ultimately the progression of atherosclerotic plaque formation. Upward and downward arrows indicate increased and decreased abundance or levels, respectively; connecting arrows indicate the direction of the illustrated processes.

Diet plays a crucial role in shaping the gut microbiome [8]. In particular, a Western-style diet high in saturated fats and low in fiber can induce dysbiosis. Under physiological conditions, the gut microbiota of both humans and mice is dominated by *Bacteroidetes* and *Firmicutes*, which together account for nearly 90% of the total microbial population. An elevated *Firmicutes*/*Bacteroidetes* (F/B) ratio has been associated with obesity and cardiovascular disease and is regarded as an important indicator of intestinal health [9]. In contrast, a typical Western diet—characterized by high saturated fat, refined sugars, and low dietary fiber—readily induces microbial dysbiosis (Figure 1). Such a diet may promote the expansion of disease-associated or potentially pro-inflammatory taxa, while reducing short-chain fatty acid (SCFA)-producing taxa associated with barrier-supporting and anti-inflammatory functions. Consequently, the impaired production of SCFAs compromises gut barrier integrity, increases intestinal permeability (commonly referred to as “leaky gut”), and facilitates the translocation of microbial components such as lipopolysaccharides (LPS) into the circulation. These processes induce systemic inflammation, a key driver of atherosclerosis [10].

### 2.1. Microbial Metabolites

Microbial metabolites represent critical mediators of the gut–heart axis, exerting systemic regulation over inflammatory responses, lipid metabolism, and immune homeostasis [11]. Dysbiosis often promotes the overgrowth of trimethylamine (TMA)-producing bacteria such as *Escherichia coli* and *Klebsiella pneumoniae*, resulting in elevated plasma levels of trimethylamine N-oxide (TMAO), which has been closely linked to increased cardiovascular risk [12]. Conversely, dysbiosis suppresses SCFA-producing microbes, thereby weakening lipid balance and anti-inflammatory defenses, ultimately dismantling the protective barrier against AS [13]. Additional key metabolites implicated in the progression of AS will be discussed in Section 3.

### 2.2. Disruption of the Intestinal Barrier

When microbial dysbiosis compromises gut barrier function, bacterial products such as LPS and peptidoglycan can translocate into circulation. These molecules interact with toll-like receptor 4 (TLR4) on immune cells, activating the NF-κB signaling pathway and inducing chronic low-grade inflammation. This cascade promotes the production of reactive oxygen species, adhesion molecules, and proinflammatory cytokines such as TNF-α, IL-1β, and IL-6. Enhanced Th1/Th17 immune responses subsequently drive endothelial injury, plaque formation, and instability [10,14]. This inflammatory axis plays a central role in both the initiation and progression of AS [9].

### 2.3. Evidence and Hypotheses Regarding Microbial Translocation to Atherosclerotic Plaques

Microbial DNA and bacterial taxa have been detected in human atherosclerotic plaques. Koren et al. [6] identified bacterial communities within atherosclerotic plaques and reported overlap with oral and gut microbiota, including taxa such as *Veillonella* and *Streptococcus*. These findings support the presence of plaque-associated microbial signals but do not establish their viability, origin, or route of entry. Potential hypotheses of microbial translocation and persistent intraplaque colonization are discussed in Section 6.4. Together, these findings support a systemic contribution of gut microbiota to atherosclerosis [10,11].

## 3. Representative Gut Microbial Metabolites in Atherosclerosis

**Figure 2:**
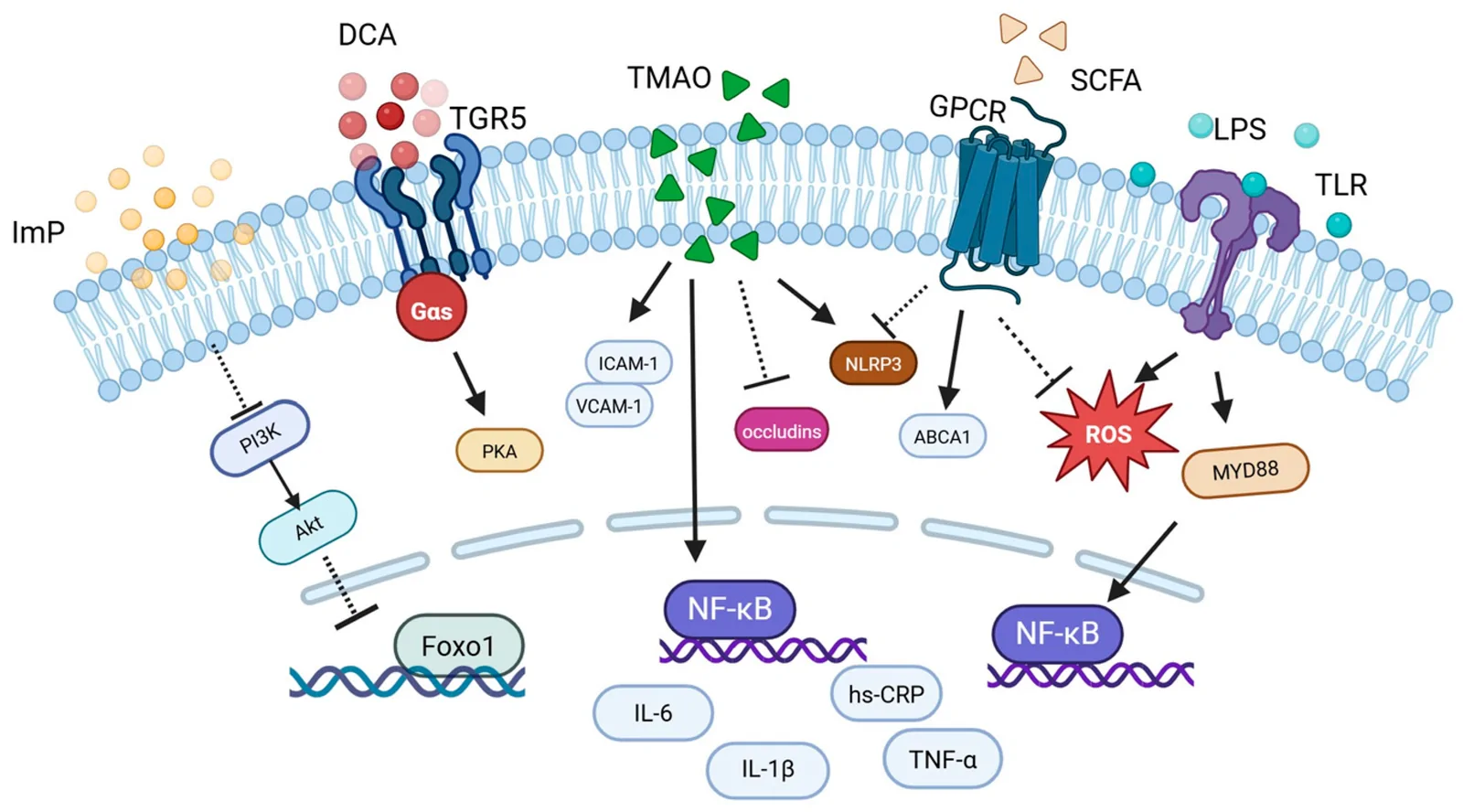
Proinflammatory and anti-inflammatory mechanisms of gut microbiota-related metabolites. Arrowheads indicate activation or promotion, whereas blunt-ended lines indicate inhibition.

The gut microbiota contributes to atherosclerosis not only through compositional alterations but also via bioactive metabolites that play pivotal roles in disease initiation and progression. Among these, TMAO and short-chain fatty acids have been most extensively studied (Figure 2).

TMAO activates the NF-κB pathway, promotes the expression of endothelial cell adhesion molecules, stimulates systemic oxidative stress, impairs tight junctions between endothelial cells, and promotes the production of inflammatory factors as well as inflammasome activation. LPS binds to Toll-like receptors and activates the NF-κB pathway via the MYD88 complex, promoting the generation of downstream inflammatory factors and simultaneously activating systemic oxidative stress. SCFAs bind to G protein-coupled receptors, which can inhibit oxidative stress and inflammasome activation in immune cells, while promoting ABCA1 protein expression in macrophages, enhancing lipid efflux, and suppressing the progression of atherosclerosis. DCA activates the TGR5 receptor and enhances the cAMP/PKA signaling pathway, thereby inhibiting NLRP3 inflammasome activation, improving metabolism, and suppressing platelet activation, exerting anti-inflammatory and antithrombotic effects. ImP inhibits the PI3K/Akt pathway and leads to sustained activation of the transcription factor FOXO1, thereby impairing endothelial cell function and reducing its migratory, proliferative, and reparative capacities. Arrowheads indicate activation or promotion, whereas blunt-ended lines indicate inhibition. Figure 2 was created with BioRender.com [15].

### 3.1. Trimethylamine N-Oxide

Nutrients such as choline and carnitine—abundant in red meat and dairy products—are metabolized by gut microbes into trimethylamine, which is subsequently oxidized by hepatic flavin-containing monooxygenases into TMAO. Experimental studies have identified several potential proatherogenic effects of TMAO. In cell-based studies, TMAO disrupted endothelial junctional proteins, including occludin, and activated MAPK/NF-κB and NLRP3 signaling pathways [16,17]. Animal studies further showed that TMAO promoted macrophage cholesterol accumulation and foam-cell formation, increased atherosclerotic lesion burden, and impaired reverse cholesterol transport [18,19]. In addition, combined human platelet, animal, and observational studies suggested that TMAO may enhance platelet reactivity and thrombosis [20]. Human evidence is predominantly observational. In patients with coronary artery disease or acute myocardial infarction, higher circulating TMAO levels have been associated with vulnerable plaque characteristics, including thinner fibrous caps, thin-cap fibroatheroma, microvessels, and plaque rupture [21,22,23].

However, circulating TMAO concentrations are also influenced by diet, renal clearance, and host comorbidities. Impaired renal function can substantially increase circulating TMAO levels, while controlled dietary studies have shown that fish- or whole-grain-rich diets may increase TMAO despite generally favorable cardiometabolic effects [24,25]. Therefore, TMAO may be considered a context-dependent biomarker associated with cardiovascular and plaque-related risk, and its interpretation should take renal function, diet, and the clinical characteristics of the study population into account.

### 3.2. Short-Chain Fatty Acids

Short-chain fatty acids (SCFAs), principally acetate, propionate, and butyrate, are produced through microbial fermentation of dietary fiber but differ in their abundance, tissue utilization, and physiological effects [26]. Acetate is generally the most abundant SCFA and is more readily available to peripheral tissues, whereas propionate is largely taken up by the liver and butyrate is preferentially utilized by colonocytes, contributing to intestinal barrier integrity and immune regulation [27,28].

Importantly, acetic acid, propionic acid and butyric acid play different roles in atherosclerosis. In ApoE^−^/^−^ mice, propionate reduced intestinal cholesterol absorption and atherosclerotic lesion formation by increasing intestinal regulatory T cells and IL-10 levels and suppressing NPC1L1 expression. The same study also included a proof-of-concept human intervention showing reductions in total and LDL cholesterol following propionate supplementation [29]. Butyrate attenuated atherosclerosis in ApoE^−^/^−^ mice and promoted ABCA1-mediated cholesterol efflux, with complementary macrophage experiments supporting this mechanism [30]. Evidence for acetate is relatively more limited; however, a recent study in ApoE^−^/^−^ mice and macrophages showed that acetate attenuated atherosclerosis through GPR43–AMPK signaling and reduced macrophage proliferation, oxidative stress, and inflammation [31]. Overall, these findings indicate that acetate, propionate, and butyrate may exert distinct effects on atherosclerosis and should therefore be considered individually.

### 3.3. Lipopolysaccharides (LPS)

LPS, a proinflammatory component released from Gram-negative bacteria, frequently enters systemic circulation under conditions of increased gut permeability, as observed in obesity, type 2 diabetes, and nonalcoholic fatty liver disease. Circulating LPS activates NF-κB signaling via TLR 4 on immune cells [32,33], leading to NADPH oxidase 2 (NOX2) activation, increased oxidized low-density lipoprotein levels, platelet activation, and macrophage-mediated inflammatory responses within plaques [34,35]. These processes compromise plaque stability and promote vulnerable plaque formation.

### 3.4. Phenylacetylglutamine (PAGln)

Phenylacetylglutamine, a gut microbial metabolite of phenylalanine, interacts with G-protein-coupled receptors—particularly adrenergic receptors—to modulate cardiovascular and platelet function [36]. Elevated plasma PAGln levels have been associated with plaque burden and in-stent restenosis and are believed to contribute to disease progression by enhancing platelet reactivity and inflammation. Thus, this metabolite may serve as a predictive biomarker for obstructive coronary artery disease and complex lesions [37,38].

### 3.5. Bile Acids (BAs)

Dysregulated bile acid metabolism is also implicated in AS. Hepatic cholesterol 7α-hydroxylase (CYP7A1) converts cholesterol into primary bile acids, which are subsequently transformed by bacterial bile salt hydrolases (BSHs) into secondary bile acids, including deoxycholic acid, lithocholic acid, and ursodeoxycholic acid. These secondary bile acids modulate AS mainly via two host receptors: the nuclear receptor farnesoid X receptor (FXR) and the bile acid receptor TGR5. Patients with AS exhibit reduced excretion of secondary bile acids [39]. Activation of hepatic FXR promotes lipoprotein clearance, lowers cholesterol, and reduces plaque formation, whereas intestinal FXR deficiency or antagonism has also been shown to attenuate AS [40,41]. TGR5 activation exerts anti-inflammatory and vasoprotective effects by suppressing NF-κB/Akt signaling, reducing cytokine production, decreasing monocyte adhesion, and enhancing endothelial nitric oxide generation [42,43].

Metabolites such as TMAO, SCFAs, LPS, PAGln, and BAs play multidimensional roles in inflammation, immunity, and metabolic regulation in AS. Targeting these metabolic pathways represents a promising avenue for the development of novel therapeutic strategies for AS prevention and treatment.

## 4. Gut Microbiota Alterations in Atherosclerosis-Related Diseases

### 4.1. Gut Microbiota Alterations in Coronary Artery Disease (CAD)

Recent studies have systematically revealed the imbalance of gut microbiota in AS and related cardiovascular and cerebrovascular diseases. A recent systematic review and meta-analysis demonstrated that in patients with CAD, the relative abundance of *Bacteroidetes* and *Lachnospiraceae* was significantly reduced, whereas *Proteobacteria* (particularly *Enterobacteriaceae*) and certain *Firmicutes* taxa (such as *Streptococcus*) were increased [44]. This microbial imbalance may weaken the anti-inflammatory and metabolic homeostatic functions of the gut, thereby exacerbating systemic inflammation and endothelial dysfunction and accelerating the onset and progression of CAD.

Further studies have correlated gut microbiota composition with the degree of coronary stenosis and clinical phenotypes. In patients with severe stenosis (≥70%), genera such as *Sutterella* and *Faecalitalea* were enriched [45]. By contrast, in mild stenosis (<50%), genera such as *Fusicatenibacter* increased, while *Olsenella* and *Anaerococcus* decreased [46]. Moreover, the relative abundance of *Sutterella* and *Bilophila* correlated positively with plaque burden on intravascular ultrasound, whereas *Faecalibacterium* was associated with stenotic area as measured by optical coherence tomography (OCT) [47]. Distinct microbiota profiles have also been observed across different CAD subtypes. For instance, patients with stable angina (SA) exhibited enrichment of genera such as *Ruminococcus*, *Duncaniella*, and *Faecalimonas*, whereas unstable angina (UA) patients showed enrichment of *Lachnospira* and *Harryflintia*. In acute myocardial infarction (AMI), elevated levels of *Alistipes* and *Rothia* were observed [48,49]. Xu et al. found that patients with acute coronary syndrome exhibited a distinct profile characterized by enrichment of pro-inflammatory microbiota (e.g., *Streptococcus*), a decreased *Prevotella*/*Bacteroides* ratio, and significantly elevated circulating levels of metabolites such as 3-hydroxybutyrate, succinate, and 2-hydroxybutyrate. These ACS-specific features showed partial reversibility after patients transitioned to a stable recovery phase following treatment [50]. Additionally, in CAD patients with diabetes, species such as *Bifidobacterium longum* and *Ruminococcus torques* were relatively abundant, whereas in CAD patients without diabetes, species such as *Alistipes putredinis* and *Roseburia inulinivorans* were more enriched [51].

### 4.2. Gut Microbiota Alterations in Atherosclerotic Carotid Artery Stenosis (CAS)

Beyond coronary disease, patients with CAS and ischemic stroke also exhibit notable gut microbiota alterations. Studies have reported enrichment of *Bacteroides eggerthii*, *Escherichia coli*, and *Klebsiella pneumoniae* in CAS patients [52]. The abundances of *Megasphera* and *Muribaculaceae* were decreased, while certain species within the *Bacteroides* genus exhibited a positive correlation with plasma lipid metabolites [53]. In patients with atherosclerotic stroke, *Bacteroidetes* were markedly decreased, while *Firmicutes*, *Proteobacteria*, and *Actinobacteria* increased, accompanied by enhanced TMAO metabolism. Plasma TMAO levels were positively correlated with carotid plaque burden [54]. Furthermore, shared bacterial genera such as *Klebsiella*, *Streptococcus*, and *Clostridium_sensu_stricto_1* were identified in both carotid plaques and fecal samples, suggesting microbial translocation across a compromised gut barrier may contribute to local plaque inflammation [55]. The gut microbiota features for various atherosclerotic diseases are presented in Table 1.

### 4.3. Gut Fungi and Atherosclerosis

Observed evidence: Notably, the gut mycobiome also appears to play a role in atherosclerosis-related diseases. Su et al. reported significant enrichment of opportunistic fungi, including *Candida albicans*, *Exophiala spinifera*, and *Malassezia restricta*, in patients with atherosclerotic cardiovascular disease (ASCVD) [56]. An et al. further observed that *Mucoromycota* and *Mortierellomycota* correlated with CAD severity [57]. Mechanistically, fungal metabolites may influence host pathways relevant to atherosclerosis. For example, *Candida albicans* produces formyl-methionine(fMet), which can activate the HIF-2α pathway and promote ceramide synthesis in related experimental settings, suggesting a potential link between fungal metabolites and proatherogenic host responses [58].

### 4.4. Hypothesis: Potential Cross-Kingdom Interactions Between Gut Fungi and Bacteria in Atherosclerosis

We hypothesize that gut fungi and bacteria may engage in cross-kingdom interactions that contribute to atherosclerosis progression. Given the enrichment of opportunistic fungi in ASCVD and the proatherogenic effects of fungal metabolites such as formyl-methionine, it is plausible that fungal expansion may cooperate with bacterial metabolic disturbances rather than acting alone. For example, fungal overgrowth may coincide with the loss of SCFA-producing bacteria associated with metabolic and barrier-supporting functions, while fungal and bacterial products may converge on shared inflammatory pathways, including HIF-2α-, ceramide-, and NF-κB-related signaling, thereby aggravating plaque development. Future studies using defined fungal–bacterial consortia in gnotobiotic models may help disentangle these cross-kingdom contributions to AS severity.

Due to the technical challenges of fungal sequencing and culture, mechanistic studies on the role of gut fungi in AS are still in their infancy. Moreover, AS is a systemic disease, and its manifestations vary significantly across different vascular beds. The risk factors, clinical outcomes, and plaque characteristics vary significantly across different types of atherosclerotic diseases [59]. Whether gut microbiota exert differential effects across AS subtypes, and whether there exist common versus disease-specific microbial taxa associated with atherosclerotic phenotypes, remains an open question.

## 5. Novel Mechanisms of Gut Microbiota in Atherosclerosis Revealed by Multi-Omics and Animal Studies

In recent years, advances in multi-omics technologies and animal models have provided novel insights into the mechanisms by which gut microbiota and their metabolites contribute to the development and progression of AS. These findings highlight the complex roles of microbiota in thrombosis, immune regulation, metabolic homeostasis, endothelial function, and intestinal barrier integrity (Table 2).

### 5.1. Modulation of Platelet Function and Thrombosis

Gut microbiota can regulate platelet function and thereby influence thrombotic risk. Compared with patients with CAD, healthy individuals were found to have increased abundance of *Bacteroides* species and elevated serum levels of deoxycholic acid (DCA). Ex vivo platelet experiments showed that DCA acts through TGR5 and activates downstream cAMP/PKA signaling to suppress agonist-induced platelet activation. Supplementation with DCA, colonization with *Bacteroides vulgatus*, or fecal microbiota transplantation from healthy donors significantly reduced platelet hyperreactivity, alleviated thrombosis, and mitigated myocardial ischemia/reperfusion injury in AS mouse models [60].

### 5.2. Maintenance of Intestinal Barrier Integrity

Gut microbiota also modulate AS progression indirectly by preserving intestinal barrier function, thereby reducing endotoxin translocation and systemic inflammation. In a cohort of 371 individuals, *Faecalibacterium prausnitzii* abundance was inversely associated with CAD. Functional experiments further showed that live *F. prausnitzii* increased ZO-1 expression in intestinal epithelial cells, while oral administration in ApoE^−^/^−^ mice strengthened intestinal mucus and tight-junction barriers, reduced circulating LPS and vascular inflammation, and attenuated atherosclerotic lesions [61].

Additional evidence has been obtained predominantly from animal models. In ApoE^−^/^−^ mice, eIF6 deficiency reshaped the gut microbiota, including enrichment of *Lactobacillus acidophilus*, and was associated with improved intestinal barrier function and reduced atherosclerotic burden; direct *L. acidophilus* supplementation further alleviated barrier dysfunction and vascular inflammation [62]. Similarly, chronic apical periodontitis aggravated atherosclerosis in high-fat diet-fed ApoE^−^/^−^ mice and was accompanied by gut dysbiosis, altered bile acid metabolism, and reduced expression of intestinal tight-junction and mucin proteins [63]. These findings support intestinal barrier dysfunction as an important mediator of microbiota–vascular interactions.

### 5.3. Microbial Virulence Factors and Metabolic Disruption

Human and multi-omics studies have identified several microbial virulence factors and metabolic pathways associated with atherosclerosis. In patients with type 2 diabetes mellitus (T2DM)-related atherosclerotic cardiovascular disease (ASCVD), *Enterobacteriaceae*/*Escherichia-Shigella* and type III secretion system (T3SS) -related virulence genes were enriched and positively associated with the severity of coronary stenosis [64]. Circulating 3-methyl-L-histidine (3-MH) levels were positively associated with carotid intima–media thickness and atherogenic lipid profiles [65].

Mechanistic experiments further clarified these associations. In human intestinal epithelial cells, T3SS-competent enterohemorrhagic Escherichia coli (EHEC) induced ferroptosis and disrupted barrier integrity, accompanied by increased FTH1 expression, whereas FTH1 knockdown attenuated these effects [64]. In Caco-2 cells, 3-MH enhanced HNF1A-dependent NPC1L1 expression and cholesterol uptake [65]. Animal experiments provided additional causal support. T3SS-competent *Citrobacter rodentium* aggravated intestinal barrier dysfunction and atherosclerotic lesions in diabetic ApoE^−^/^−^ mice [64]. In ApoE^−^/^−^ mice, direct administration of 3-MH increased intestinal cholesterol absorption and plaque burden [65]. These studies support several microbiota-dependent proatherogenic pathways, although their direct causal roles in human atherosclerosis remain to be established.

### 5.4. Modulation of Endothelial and Immune Cell Function

In human cohorts, circulating imidazole propionate (ImP) levels were elevated in patients with CAD [66], whereas indole-3-propionic acid (IPA) levels were reduced and were associated with ASCVD severity [67]. In addition, patients with ASCVD showed reduced abundance of *Bacteroides ovatus* and lower circulating levels of indole-3-acetic acid (IAA) [68].

Mechanistic studies further revealed distinct effects of these metabolites on vascular and immune cells. In human aortic endothelial cells, ImP impaired endothelial migration and angiogenic capacity by suppressing PI3K/AKT signaling and sustaining FOXO1 activation [66]. In human- and mouse-derived macrophages, IPA promoted cholesterol efflux through the miR-142-5p–ABCA1 pathway [67]. IAA inhibited TLR4/MyD88/NF-κB signaling in proinflammatory macrophages while promoting M2 macrophage polarization [68].

Animal studies further supported these mechanisms. ImP impaired vascular repair and increased atherosclerotic lesion burden and macrophage accumulation in ApoE^−^/^−^ mice [66]. IPA supplementation enhanced macrophage reverse cholesterol transport and attenuated plaque formation [67]. Similarly, *B. ovatus* or IAA intervention reduced vascular inflammation and atherosclerotic burden and improved the imbalance of M1/M2 macrophage polarization [68]. These findings further indicate that gut microbiota-derived metabolites may contribute to atherosclerosis by modulating endothelial function and macrophage inflammatory responses.

Through intertwined mechanisms—including platelet regulation, barrier preservation, metabolic reprogramming, and modulation of endothelial and immune cell functions—the gut microbiota exerts systemic influence on vascular inflammation and plaque dynamics. These findings extend our understanding of AS pathogenesis and highlight novel therapeutic opportunities targeting host–microbiota interactions.

## 6. Advances in the Study of Intraplaque Microorganisms

### 6.1. Microbial Composition of Atherosclerotic Plaques and Potential Sources

Evidence for microorganisms within atherosclerotic plaques spans several levels, although most human studies remain based on microbial DNA detection. Using 16S rRNA gene sequencing, metagenomic sequencing, or quantitative PCR, previous studies have identified bacterial genetic material in carotid and coronary plaques, including *Streptococcus*, *Veillonella*, *Porphyromonas*, and *Neisseria* [6,69,70,71,72]. These approaches demonstrate the presence of microbial DNA but cannot by themselves establish microbial viability, metabolic activity, or functional involvement in plaque biology.

Spatial localization provides an additional level of evidence. Huang et al. directly visualized *Porphyromonas gingivalis*-positive clusters in human coronary plaques using a Pg-specific FISH probe [7]. Karhunen et al. detected viridans group streptococcal DNA by quantitative PCR and further localized streptococcal immunoreactivity within the atheromatous core, fibrous cap, and plaque rupture sites by immunohistochemistry [73]. These findings strengthen the evidence for localized microbial signals within defined plaque regions.

Direct evidence of microbial viability in human plaques remains scarce. *Chlamydia pneumoniae* mRNA has been detected by reverse-transcription PCR in subsets of carotid plaques, supporting possible microbial viability in selected specimens [74,75]. In addition, Lanter et al. cultured viable *Cutibacterium acnes* and several other bacterial species from human carotid atherosclerotic tissue [76]. However, such RNA- and culture-based evidence remains limited and is particularly susceptible to contamination in low-biomass vascular specimens.

Several studies have also investigated the potential oral or intestinal origin of plaque-associated microbial signals using DNA-based approaches. Sato et al. initially detected bacterial DNA by 16S rRNA gene PCR and subsequently used 16S rRNA gene sequencing to compare carotid and dental plaques, reporting substantial overlap between the two microbial communities [71]. Lv et al. performed V3–V4 16S rRNA gene sequencing of carotid plaque and fecal samples and identified 11 shared bacterial genera [55]. Figuero et al. used quantitative and nested PCR to detect periodontal pathogen DNA in vascular, blood, and subgingival samples [72]. These findings support a possible oral or intestinal contribution to plaque-associated microbial signals, but DNA overlap alone cannot establish direct microbial translocation or persistent colonization (Table 3).

### 6.2. Sources of Heterogeneity in Intraplaque Microbiome Studies

Reported intraplaque microbial signatures vary considerably across studies, likely reflecting both biological and methodological heterogeneity. First, atherosclerotic plaques differ across vascular beds. Rupture-prone coronary plaques are typically characterized by lipid-rich necrotic cores and thin fibrous caps, whereas plaques in peripheral arteries, particularly the femoropopliteal arteries, tend to contain more fibrotic components, less lipid, and fewer inflammatory cells [77,78,79]. Local hemodynamic conditions also differ among vascular beds [80]. We therefore speculate that differences in plaque composition and surrounding hemodynamic environments may contribute to differential microbial colonization. Patient-related factors, including age, medical history, and medication exposure, may further influence plaque-associated microbial signals.

Second, plaque sampling strategies vary considerably. Many studies analyze whole or bulk plaque specimens without clearly specifying the exact intraplaque region sampled, which may limit comparability between studies [6,69,70,71,72]. Third, differences in microbial detection and analytical methods may also contribute to discordant microbial profiles [69,70,81]. The specific methodological differences between 16S rRNA sequencing and shotgun metagenomics are discussed in detail below. Finally, contamination control remains an important concern. Atherosclerotic plaques are low-biomass specimens and are therefore particularly susceptible to contamination during surgical sampling, specimen handling, DNA extraction, and sequencing [82]. However, sampling procedures and contamination-control strategies are not consistently described across studies [83,84]. Collectively, these biological, sampling, methodological, and contamination-related factors may partly explain the heterogeneous intraplaque microbial signatures reported across studies.

### 6.3. Functional Roles of Intraplaque Microorganisms

Beyond microbial identification, recent studies have begun to explore how intraplaque microorganisms influence plaque biology, particularly through interactions with immune cells. Jing et al. first demonstrated that probiotic-derived membranous vesicles (MVs) from *Lactobacillus rhamnosus* could selectively target foam macrophages, activate the NR1H3–ABCA1 pathway to promote cholesterol efflux, and polarize macrophages from pro-inflammatory M1 (CD86^+^) to anti-inflammatory M2 (CD206^+^) phenotypes. These effects reduced lipid deposition and enhanced plaque stability in animal models [85]. Conversely, periodontitis-associated oral bacteria such as *Fusobacterium nucleatum* were shown to enter the vascular intima via the bloodstream, inducing macrophage M1 polarization and lipid metabolic dysregulation [86]. Similarly, *P. gingivalis* promoted macrophage lipid uptake and plaque instability by activating TLR4 and MAPK/ERK–FOXO3 pathways, while its LPS and gingipains triggered oxidative stress-dependent necroptosis, thereby enlarging necrotic cores and weakening plaque integrity [7].

### 6.4. Hypothesis: Potential Gut–Oral–Intraplaque Crosstalk and Local Microbial Metabolism

**Figure 3:**
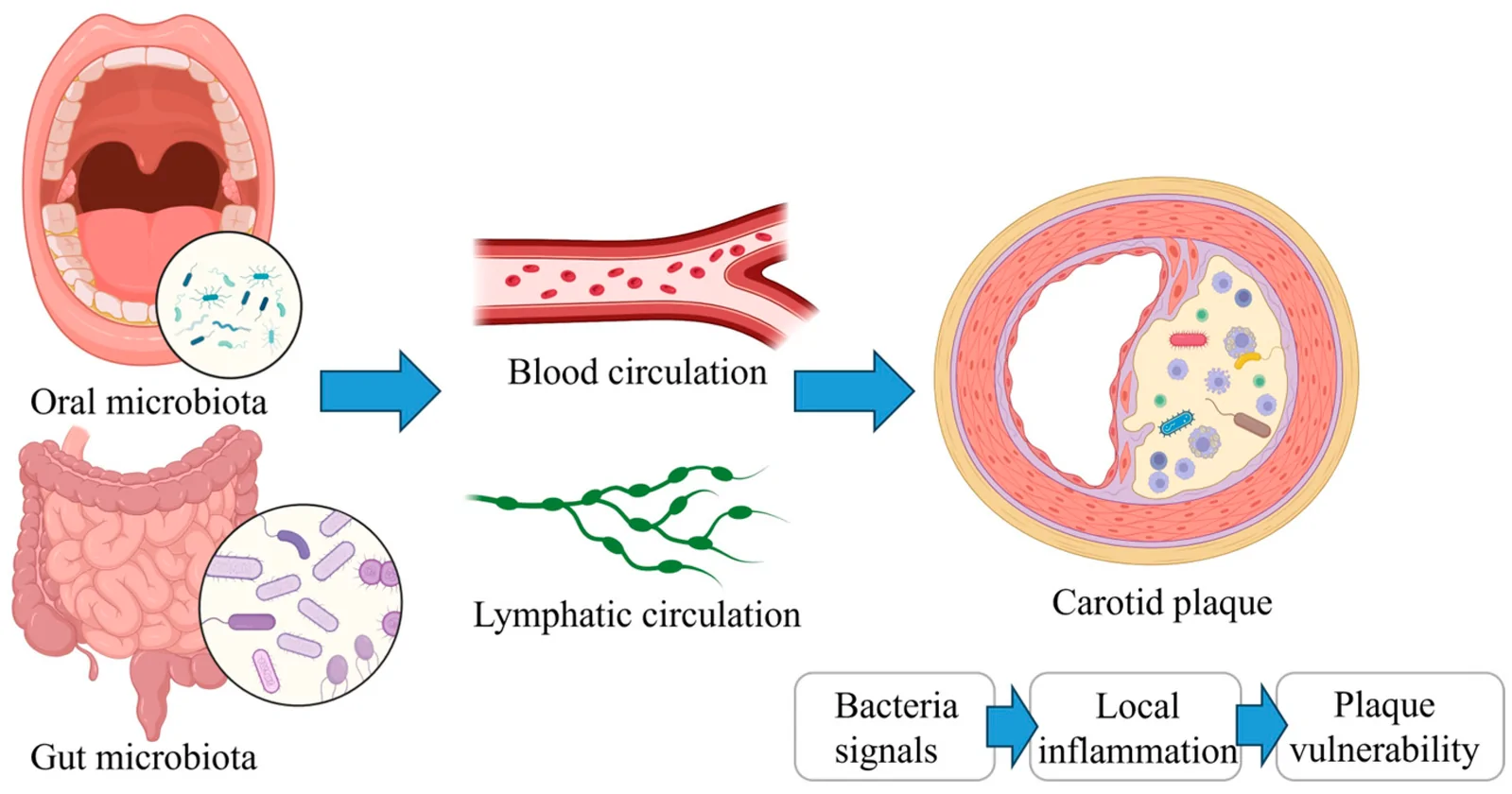
Proposed gut–oral–intraplaque microbial axis and its potential contribution to plaque vulnerability. Oral and gut microbiota may reach atherosclerotic plaques via the circulation, where bacterial signals may promote local inflammation and plaque vulnerability. This pathway remains hypothetical and requires further validation. Arrows indicate hypothesized routes of microbial translocation and potential downstream effects.

Taken together, previous studies have reported overlapping bacterial taxa across oral, gut, blood, and atherosclerotic plaque samples [6,55,71,72]. Experimental studies in ApoE^−^/^−^ mice further support the biological plausibility of this connection, showing that *Porphyromonas gingivalis* can invade aortic tissue following chronic oral infection [87], while chronic apical periodontitis can alter gut microbial composition and impair intestinal barrier function [63]. Based on these observations, we hypothesize that some plaque-associated microorganisms may originate from disrupted oral or intestinal barriers, reach atherosclerotic lesions via hematogenous and potentially lymphatic routes, and persist within the plaque microenvironment (Figure 3).

If viable microorganisms persist locally, they may also create a plaque-specific microbial metabolic microenvironment capable of influencing plaque progression. Experimental studies suggest that periodontal pathogens reaching atherosclerotic lesions can modulate macrophage inflammation and lipid metabolism [7,86], while metagenomic analysis identified an increased abundance of sulfur-associated bacterial families in symptomatic plaques [70]. However, direct evidence demonstrating the in situ production of microbial metabolites within human atherosclerotic plaques is currently lacking. Therefore, both the proposed gut–oral–intraplaque crosstalk and local microbial metabolic activity remain working hypotheses that require further validation.

## 7. Gut Microbiota-Mediated Therapeutic Strategies Against Atherosclerosis

In recent years, a growing body of evidence has demonstrated that pharmacological agents, probiotics, natural products, and dietary interventions can modulate the gut microbiota and its metabolites, thereby improving systemic metabolism and inflammatory status and ultimately exerting protective effects against AS (Table 4).

### 7.1. Probiotics, Prebiotics, Postbiotics, and Synbiotics

Probiotics comprise live microorganisms that confer a health benefit on the host when administered in adequate amounts, prebiotics provide selectively utilized substrates, postbiotics deliver inanimate microorganisms or their components, and synbiotics combine live microorganisms with microbial substrates to achieve complementary or synergistic effects [88,89,90,91]. Accumulating evidence, derived predominantly from preclinical studies, suggests that these microbiota-directed interventions may influence atherosclerosis through multiple microbiota-dependent mechanisms [92].

#### 7.1.1. Probiotics

Probiotics are live microorganisms that confer health benefits on the host when administered in adequate amounts [88]. Several probiotic strains have shown anti-atherosclerotic potential through different levels of experimental evidence. In animal models, *Escherichia coli* Nissle 1917 attenuated atherosclerosis by reshaping the gut microbiota and serum metabolome and suppressing homocitrulline-associated Caspase1/NLRP3/GSDMD-mediated pyroptosis [93]. *Bifidobacterium animalis* subsp. lactis F1-7 reduced plaque and lipid accumulation in ApoE^−^/^−^ mice by modulating bile acid metabolism and downregulating intestinal FXR signaling [94]. By contrast, *Faecalibacterium prausnitzii* has been supported by human multi-omics, cellular, and animal evidence: reduced abundance was associated with CAD, live bacteria increased ZO-1 expression in intestinal cells, and oral supplementation strengthened intestinal barrier integrity and attenuated atherosclerosis in ApoE^−^/^−^ mice [61]. These findings support the potential of probiotics as microbiota-directed interventions.

#### 7.1.2. Prebiotics

Current evidence for the anti-atherosclerotic effects of the prebiotics summarized here is derived entirely from animal studies. In high-fat diet-fed ApoE^−^/^−^ mice, both short- and long-chain inulin reduced atherosclerotic plaque burden, inflammation, and intestinal barrier dysfunction, with short-chain inulin showing greater efficacy. These effects were accompanied by alterations in gut microbial composition and the expression of lipid metabolism-related genes, including SREBP1, PCSK9, CYP7A1, ABCA1, and ABCG1 [95]. In a separate study of male ApoE^−^/^−^ mice fed a high-fat, high-cholesterol diet, manno-oligosaccharides derived from cassia seed gum reduced atherosclerotic lesions and systemic and vascular inflammation while improving intestinal barrier integrity and reshaping the gut microbiota [96]. Similarly, aged garlic oligosaccharides attenuated atherosclerosis in high-fat, high-cholesterol diet-fed ApoE^−^/^−^ mice, accompanied by reduced circulating TMAO, increased fecal short-chain fatty acids, and alterations in predicted microbial pathways, including ABC transporter and lipopolysaccharide biosynthesis pathways [97]. Collectively, these findings provide preclinical evidence that prebiotics may influence atherosclerosis through microbiota-associated metabolic, inflammatory, and intestinal barrier pathways.

#### 7.1.3. Postbiotics

Postbiotics are preparations of inanimate microorganisms and/or their components that confer a health benefit on the host. Emerging evidence suggests that postbiotics may have therapeutic potential in atherosclerosis [90]. In an atherosclerotic mouse model induced by a high-fat diet, vitamin D3, and choline, postbiotics derived from *Weizmannia coagulans* JA845 reduced plaque formation and vascular inflammation, accompanied by lower TMAO levels, suppression of JAK/STAT3 signaling, and improved endothelial function [98]. Preclinical studies of pasteurized *Akkermansia muciniphila* further showed improvements in insulin resistance and dyslipidemia in high-fat diet-fed mice, while its outer-membrane protein Amuc_1100 interacted with TLR2 and improved intestinal barrier function [99]. Translational evidence has subsequently emerged from a randomized, double-blind, placebo-controlled study in overweight and obese insulin-resistant individuals, in which pasteurized *A. muciniphila* improved insulin sensitivity and reduced plasma total cholesterol [100]. Overall, postbiotics represent a promising microbiota-directed strategy, although direct clinical evidence for atherosclerosis remains limited.

#### 7.1.4. Synbiotics

Synbiotics combine live microorganisms with selectively utilized substrates and may provide complementary or synergistic effects on the gut microbiota [91]. In a high-cholesterol diet-induced atherosclerotic animal model, supplementation with *Lactiplantibacillus plantarum* M10 and isomaltose improved lipid metabolism and gut microbial composition, reduced aortic thickening, and attenuated vascular inflammation, accompanied by decreased NF-κB signaling and lower expression of IL-6, TNF-α, MCP-1, and ICAM-1 [101]. Limited clinical evidence from randomized trials in patients with coronary heart disease or dyslipidemia has shown improvements in inflammatory and oxidative-stress markers, nitric oxide, TMAO, and endotoxin levels, although carotid intima–media thickness was unchanged [102,103]. These findings suggest that synbiotics may influence atherosclerosis-related metabolic and inflammatory pathways, although evidence for their effects on plaque progression or cardiovascular events remains limited.

### 7.2. Traditional Chinese Medicine (TCM) and Natural Products

Evidence for microbiota-mediated effects of TCM and natural products is predominantly preclinical. Among the available studies, puerarin has been evaluated in both experimental models and humans. In ApoE^−^/^−^ mice, puerarin attenuated atherosclerosis partly by reducing *Prevotella copri* and its TMA-producing capacity, while a short-term intervention in patients with carotid plaques reduced *P. copri* abundance and circulating TMAO levels [104]. Most other findings are derived from animal and mechanistic studies. Silybin, palmatine and aucubin attenuated atherosclerotic lesions in experimental models through modulation of gut microbial composition and metabolites, intestinal barrier function, lipid metabolism, and vascular inflammation [105,106,107]. Buyang Huanwu Decoction similarly reduced plaque burden and improved plaque-related features in ApoE^−^/^−^ mice, accompanied by alterations in gut microbiota, reduced TMAO levels, altered bile acid metabolism, and suppression of TLR4/NF-κB/NLRP3 signaling [108]. Shenxiang Suhe pill has also been shown to modulate gut microbiota and metabolites and improve cardiac function [109].

### 7.3. Dietary Grains and Other Interventions

Evidence for these dietary and natural interventions is also predominantly pre-clinical. In ApoE^−^/^−^ mice, whole-grain highland barley reduced atherosclerotic plaque burden and modulated gut microbiota, accompanied by suppression of NLRP3-related inflammation [110], while lingonberry supplementation reduced plaque formation and altered gut microbial composition and metabolites [111]. Foxtail millet bran-derived FMB-BBTI similarly attenuated atherosclerosis and remodeled the gut microbiota in ApoE^−^/^−^ mice [112]. Other interventions have been supported by animal and mechanistic studies: indole-3-carbinol reduced atherosclerosis in high-choline-fed ApoE^−^/^−^ mice and inhibited lipid accumulation in macrophages [113], whereas quercetin modulated gut microbial and metabolic profiles in experimental atherosclerosis [114]. *Edgeworthia gardneri* extract reduced atherosclerosis in ApoE^−^/^−^ mice, with antibiotic depletion and fecal microbiota transplantation supporting a microbiota-dependent improvement in intestinal barrier integrity [115].

Although these therapeutic strategies differ in their molecular targets, many converge on microbiota–host interactions [92]. This suggests that future AS therapies should adopt a multidimensional approach, combining drugs, probiotics, and dietary interventions to precisely modulate the “gut–circulation–vascular” axis. However, several challenges limit the translation of microbiota-targeted interventions into clinical practice. Substantial interindividual variation in baseline gut microbiota, habitual diet, medication exposure, and host characteristics may influence microbial colonization and treatment responses, thereby contributing to heterogeneous efficacy and limited reproducibility across studies [116]. Moreover, the effects of probiotics and related microbiota-directed interventions are often strain-, formulation-, and context-specific, while the optimal dose, treatment duration, combination strategy, and manufacturing standards remain insufficiently standardized [117]. Although commonly used probiotics are generally well tolerated, safety should be evaluated according to the specific strain and target population, particularly in severely ill or immunocompromised individuals, and long-term safety data remain limited [118]. Importantly, most evidence for microbiota-targeted therapies in atherosclerosis remains derived from animal and mechanistic studies, whereas human trials are relatively few and mainly assess metabolic or inflammatory surrogate endpoints [119]. Large, well-designed clinical trials with standardized interventions and clinically relevant cardiovascular outcomes are therefore required before these strategies can be translated into routine clinical practice.

**Table 4:** Evidence levels of representative microbiota-targeted therapeutic strategies in atherosclerosis.

Intervention	Category	Evidence Type	Model/Population	Main Findings
Puerarin [104]	TCM/natural product	Animal + human intervention	ApoE^−/−^ mice; patients with carotid plaques	Reduced Prevotella copri abundance and TMA production; decreased circulating TMAO after short-term human intervention; attenuated atherosclerosis in ApoE^−/−^ mice.
Palmatine [105]	Natural product	Animal + microbial culture + cell	ApoE^−/−^ mice; bacterial culture; THP-1 cells	Modulated gut microbiota and microbial phenylalanine metabolism, altered inflammatory responses, and reduced atherosclerotic plaque burden.
Silybin [106]	Natural product	Animal + mechanistic	ApoE^−/−^ mice; microbiota/metabolite analyses	Increased microbiota-derived butyrate and attenuated atherosclerosis, accompanied by improvement of intestinal barrier and vascular inflammatory responses.
Aucubin [107]	Natural product	Animal + FMT + mechanistic/metabolomics	ApoE^−/−^ mice; FMT; metabolomics	Altered gut microbiota and tryptophan-related microbial metabolism, inhibited endothelial–mesenchymal transition, and attenuated atherosclerosis.
Buyang Huanwu Decoction [108]	TCM formula	Animal	ApoE^−/−^ mice	Altered gut microbiota, reduced TMAO, modulated bile acid metabolism, and suppressed TLR4/NF-κB/NLRP3 signaling, with reduced plaque burden and improved plaque-related features.
Faecalibacterium prausnitzii [61]	Next-generation probiotic candidate	Human observational + animal + cell	371 participants; ApoE^−/−^ mice; intestinal epithelial cells	Lower abundance was associated with CAD in humans; live *F. prausnitzii* increased ZO-1 expression in vitro, while oral supplementation improved intestinal barrier function and attenuated atherosclerosis in mice.
*Escherichia coli* Nissle 1917 [93]	Probiotic	Animal + mechanistic cell validation	Atherosclerotic mouse model; endothelial-cell experiments	Remodeled gut microbiota and serum metabolites and attenuated homocitrulline-associated Caspase-1/NLRP3/GSDMD pyroptosis and atherosclerosis.
Inulin [95]	Prebiotic	Animal	HFD-fed ApoE^−/−^ mice	Improved gut microbiota, intestinal barrier function, lipid metabolism, and inflammation; short-chain inulin showed greater efficacy and reduced plaque burden.
Weizmannia coagulans JA845 postbiotics [98]	Postbiotic	Animal + mechanistic/multi-omics	HFD + vitamin D3 + choline-induced atherosclerotic mice	Reduced TMAO, vascular inflammation, and plaque formation, accompanied by suppression of JAK/STAT3 signaling and improved endothelial function.
Multispecies synbiotic + inulin [102]	Synbiotic	Human randomized controlled trial	60 patients with overweight, diabetes, and coronary heart disease	Reduced inflammatory and oxidative-stress markers and increased nitric oxide; carotid intima–media thickness was not significantly improved.
Whole-grain highland barley [110]	Dietary intervention	Animal	ApoE^−/−^ mice	Remodeled gut microbiota and suppressed NLRP3-related inflammation, accompanied by reduced atherosclerotic plaque burden.
Lingonberry [111]	Dietary intervention	Animal	ApoE^−/−^ mice	Altered gut microbiota and microbial metabolites and reduced atherosclerotic plaque formation.

Abbreviations: ApoE^−/−^, apolipoprotein E-deficient; CAD, coronary artery disease; FMT, fecal microbiota transplantation; GSDMD, gasdermin D; HFD, high-fat diet; JAK, Janus kinase; NF-κB, nuclear factor kappa B; NLRP3, NOD-like receptor family pyrin domain-containing 3; TCM, traditional Chinese medicine; TLR4, Toll-like receptor 4; TMA, trimethylamine; TMAO, trimethylamine N-oxide.

## 8. Methodological Limitations and Translational Challenges in Microbiome–Atherosclerosis Research

### 8.1. Biological Heterogeneity and Clinical Confounding Factors

Biological and clinical factors can substantially influence the gut microbiome and thereby confound its association with atherosclerotic disease. Diet is particularly important because it determines the substrates available for microbial metabolism and consequently affects the production of microbial metabolites, including short-chain fatty acids (SCFAs), trimethylamine (TMA), bile acids, and tryptophan-derived metabolites [60,61,120].

Age is another important source of variation. The composition and function of the gut microbiome change throughout the lifespan, while ageing is also accompanied by immunosenescence, chronic low-grade inflammation, gastrointestinal physiological changes, and alterations in diet and medication exposure, all of which may further reshape the gut microbial ecosystem [121].

Sex may also contribute to interindividual variation in the gut microbiome. Biological sex, sex hormones, immune regulation, and sex-related lifestyle exposures may interact with the gut microbiota and microbial metabolic pathways relevant to cardiovascular disease. The relationship between estrogen and the gut microbiome is bidirectional: estrogen can influence microbial ecology and the host immune environment, whereas gut microbial β-glucuronidases participate in the deconjugation and enterohepatic recycling of estrogens, thereby affecting circulating estrogen levels [122,123]. Therefore, changes in hormonal status, such as menopause, may simultaneously influence cardiovascular risk and the gut microbiome.

Obesity, insulin resistance, and diabetes represent additional important confounding factors through more specific microbiota-related metabolic pathways. For example, insulin resistance has been associated with increased microbial biosynthetic potential for branched-chain amino acids (BCAAs), while the microbiota-derived metabolite imidazole propionate is elevated in patients with type 2 diabetes and can impair insulin signaling through the p38γ–p62–mTORC1 pathway [124,125]. Therefore, some microbial or metabolic differences observed in patients with atherosclerosis may reflect coexisting metabolic abnormalities rather than atherosclerosis itself.

Medication exposure represents another important confounding factor. Statins, which are widely used in cardiovascular populations, may themselves reshape the gut microbiome; for example, atorvastatin treatment has been associated with reduced *Clostridium* abundance and altered fecal and circulating bile acid profiles [126]. Antibiotic exposure can directly perturb susceptible microbial populations and may have long-lasting effects on microbial diversity and community composition [127].

Geography and lifestyle may further shape the baseline microbiome through differences in diet, environment, urbanization, physical activity, and other exposures. Consequently, apparently atherosclerosis-associated microbial signatures may be influenced by multiple factors, including age, sex, metabolic comorbidities, medications, and lifestyle, all of which should be adequately considered in study design and statistical analyses [128,129].

### 8.2. Methodological and Analytical Limitations of Microbiome Profiling

Methodological heterogeneity represents an additional source of variability. Differences in sample collection and storage, DNA extraction, library preparation, sequencing platform, sequencing depth, and bioinformatic processing can introduce substantial batch effects and alter microbial profiles obtained from otherwise comparable samples [130]. For 16S rRNA sequencing, primer selection and the targeted hypervariable region can preferentially amplify or underrepresent particular taxa, while the limited sequence length generally constrains taxonomic resolution, especially at the species and strain levels [131]. Sequencing depth further affects the detection of low-abundance taxa and estimates of microbial richness. By contrast, shotgun metagenomic sequencing avoids targeted 16S amplification and generally provides higher taxonomic resolution and more direct characterization of microbial gene content, although it remains influenced by sequencing depth, host-DNA contamination, reference genome coverage, and classification algorithms [132]. Thus, microbial profiles generated using different sequencing strategies should not be considered directly interchangeable.

Analytical approaches may further contribute to inconsistent findings. Microbiome sequencing data are inherently compositional; therefore, an apparent increase in the relative abundance of one taxon may partly reflect changes in other taxa rather than an absolute increase in microbial load [133]. Different differential-abundance methods can also identify markedly different sets of significant taxa from the same dataset [134]. Functional interpretation requires similar caution: pathways inferred from 16S rRNA data are predictions based on reference genomes rather than direct measurements of microbial genes or activity, and their ability to reproduce disease-associated functional changes may be limited [135]. Shotgun metagenomics can more directly characterize functional gene potential, whereas meta-transcriptomics and metabolomics provide additional information on microbial activity and metabolic output. Overall, the limited overlap in microbial signatures across atherosclerosis studies may reflect both genuine biological heterogeneity and technical or analytical variability, emphasizing the need for standardized sampling, sequencing, quality-control, and analytical procedures.

## 9. Conclusions and Perspectives

Alterations in gut microbiota composition are increasingly recognized as an important feature of cardiovascular and cerebrovascular diseases and may be influenced by diet, geography, medication exposure, and analytical methodologies. Current evidence indicates that gut dysbiosis contributes to atherosclerosis through multiple interconnected mechanisms, including altered microbial metabolite production, impairment of intestinal barrier integrity, and dysregulated immune and inflammatory responses. In parallel, the emerging detection of microbial signals within atherosclerotic plaques suggests that intraplaque microorganisms may further influence local immune activation, lipid handling, and plaque vulnerability. Together, these findings support a model in which microbiota contribute to atherosclerosis through both systemic and local mechanisms.

Despite these advances, the field is still evolving from correlative observations to mechanistic and causal investigation. Future studies should prioritize the identification of disease-associated or potentially proatherogenic taxa, clarification of host–microbe interaction pathways, and discrimination of true intraplaque colonization from contamination or transient microbial signals. Integrated multi-omics approaches, spatial metabolomics and transcriptomics, microbial viability assays, and experimental models will be essential to define the biological relevance of plaque-associated microorganisms. In addition, although microbiota-modulating therapies using drugs, probiotics, nutraceuticals, and dietary interventions show promising anti-atherosclerotic potential, their clinical translation will require large-scale, multicenter validation and standardized analytical methods.

Overall, integrating systemic microbiota–host interactions with local plaque-associated microbial signals may provide a more comprehensive framework for understanding microbiome involvement in atherosclerosis. Targeting the microbiota–host interaction network therefore offers promising avenues for innovative strategies in cardiovascular disease prevention and treatment.

## Figures and Tables

**Table 1:** Gut microbiota alterations in different cardiovascular diseases. (↑ Enrichment/↓ Reduction).

Study	Patient Groups	Methods	Gut Microbial Changes (↑ Enrichment/↓ Reduction)	Key Findings/Conclusions
Tian et al. [51]	CAD with or without T2DM vs. control	Metagenomics	CAD + T2DM: ↑ *Bifidobacterium longum*, ↑ *B. catenulatum,* ↑ *Ruminococcus torques.* CAD only: ↑ *Alistipes putredinis*, ↑ *Roseburia inulinivorans*	CAD + T2DM patients showed unique gut microbial and metabolic profiles
Nakajima et al. [47]	Gut microbiota characteristics associated with different coronary plaque phenotypes	16S rRNA	Unstable phenotype: ↑ *Dysgonomonas*, ↑ *Paraprevotella*, ↑ *Succinatimonas*, ↑ *Bacillus*. Protective phenotype: ↑ *Alistipes*, ↑ *Anaerofustis*, ↑ *Anaerostipes*, ↑ *Butyricicoccus*, ↑ *Christensenella*, ↑ *Dielma*, ↑ *Fusicatenibacter*	Specific gut taxa correlated with plaque vulnerability
Choroszy et al. [44]	CAD vs. control	Systematic review & meta-analysis	CAD: ↓ *Bacteroidetes*, ↓ *Lachnospiraceae*; ↑ *Enterobacteriaceae*, ↑ *Lactobacillus*, ↑ *Streptococcus*	Gut microbial diversity decreased in CAD; dysbiosis linked to inflammation and metabolic imbalance
Zhang et al. [48]	MCS/SA/UA/AMI vs. control	16S rRNA	MCS: ↑ *Monoglobus*, ↑ *Flavonifractor*, ↑ *Tepidibaculum*, ↑ *Eisenbergiella*, ↑ *Vampirovibrio*. SA: ↑ *Ruminococcus*, ↑ *Duncaniella*, ↑ *Rothia*, ↑ *Faecalimonas*, ↑ *Aggregatibacter*, ↑ *Holdemanella*. UA: ↑ *Lachnospira*, ↑ *Fusicatenibacter*, ↑ *Harryflintia*. AMI: ↑ *Alistipes*, ↑ *Ruminococcus*, ↑ *Flintibacter*, ↑ *Gemmiger*, ↑ *Eisenbergiella*	Distinct gut taxa may support early AMI diagnosis
Chen et al. [45]	CAD vs. control	16S rRNA sequencing	CAD: ↓ *Blautia*, ↓ *Fusicatenibacter*, ↓ *Monoglobus*, ↓ *Eubacterium*; ↑ *Sutterella*, ↑ *Lachnospiraceae*, ↑ *Collinsella*, ↑ *Faecalitalea*	Dysbiosis interacted with host genetics, elevating CAD risk
Dong et al. [49]	AMI vs. stable CAD vs. control	16S rRNA + metagenomics	AMI: ↑ *Alistipes*, ↑ *Streptococcus*, ↑ *Ruminococcus*, ↑ *Lactobacillus*, ↑ *Faecalibacterium*. Stable CAD: ↑ *Butyricicoccus*, ↑ *Prevotella*	Gut microbiota–metabolite models distinguished AMI from stable CAD
Wang et al. [46]	Coronary stenosis <50% vs. ≥50%	16S rRNA	<50% stenosis: ↑ *Fusicatenibacter*; ↓ *Olsenella*, ↓ *Anaerococcus*. ≥50% stenosis: ↑ *Desulfovibrio*, ↑ *Moraxella*, ↑ *Actinomyces*; ↓ *Ilumatobacter*, ↓ *Aeromonas*, ↓ *Litorilinea*, ↓ *Loktanella*	Gut microbiota profiles correlated with stenosis severity
Xu et al. [54]	LAA stroke vs. CE stroke vs. control	16S rRNA	LAA stroke: ↑ *Parabacteroides*, ↑ *Klebsiella*, ↑ *Desulfovibrio*, ↑ *Enterococcus*, ↑ *Eubacterium*, ↑ *Lactobacillus*, ↑ *Bifidobacterium*	LAA stroke patients had distinct dysbiotic profiles; some taxa may serve as biomarkers
Chen et al. [52]	CAS vs. control	Shotgun metagenomic sequencing	CAS: ↑ *Bacteroides eggerthii*, ↑ *E. coli*, ↑ *Klebsiella pneumoniae*, ↑ *Acidaminococcus*, ↑ *Gemella*, ↑ *Streptococcus anginosus*, ↑ *Turicibacter*. Control: ↑ *Prevotella copri*, ↑ *Parabacteroides,* ↑ *Haemophilus parainfluenzae*, ↑ *Bacteroides* sp. 3_1_19	CAS-enriched taxa correlated with inflammation and lipid levels; TMAO/LPS pathways upregulated
Zhang et al. [53]	ICAS vs. control	16S rRNA	ICAS: ↑ *Bacteroidetes*, ↑ *Bacteroides*, ↑ *Parabacteroides*; ↓ *Megasphaera*, ↓ Muribaculaceae	ICAS patients showed significant gut microbial and metabolic alterations

Abbreviations: CAD, coronary artery disease; T2DM, type 2 diabetes mellitus; MCS, mild coronary stenosis; SA, stable angina; UA, unstable angina; AMI, acute myocardial infarction; LAA, large-artery atherosclerosis; CE, cardioembolism; CAS, carotid artery stenosis; ICAS, intracranial atherosclerotic stenosis.

**Table 2:** Representative Human, In Vitro/Ex Vivo, and Animal Evidence for Gut Microbiota-Related Mechanisms in Atherosclerosis and Vascular Dysfunction.

Study	Pathway	Human Observational Evidence	In Vitro/Ex Vivo Evidence	Animal Evidence
Koay et al. [21]	TMAO–plaque vulnerability	Plasma TMAO was not associated with coronary artery calcium or carotid intima–media thickness in the Framingham Heart Study.	—	In a tandem-stenosis mouse model, plasma TMAO was associated with markers of plaque instability.
Haghikia et al. [29]	Propionate–Treg/IL-10–NPC1L1	Propionate supplementation reduced total and LDL cholesterol in a proof-of-concept human study	—	Propionate increased intestinal Tregs and IL-10, suppressed NPC1L1 expression, reduced cholesterol absorption, and attenuated atherosclerosis in ApoE^−^/^−^ mice
Du et al. [30]	Butyrate–ABCA1–cholesterol efflux	—	Butyrate promoted ABCA1-mediated cholesterol efflux in macrophages	Butyrate increased ABCA1 expression and attenuated atherosclerosis in ApoE^−^/^−^ mice
Liu et al. [37]	PAGln–coronary atherosclerosis	Higher circulating PAGln was associated with greater coronary atherosclerotic severity	—	—
Qi et al. [60]	*B. vulgatus*–DCA–TGR5	Reduced *B. vulgatus* abundance and serum DCA in CAD; lower DCA was associated with greater platelet reactivity	DCA inhibited platelet activation and aggregation via TGR5–cAMP/PKA signaling	DCA, *B. vulgatus*, or healthy-donor FMT reduced platelet hyperreactivity, thrombosis, and myocardial I/R injury in ApoE^−^/^−^ mice
Yang et al. [61]	*F. prausnitzii*–intestinal barrier	Lower *F. prausnitzii* abundance was associated with CAD	Live *F. prausnitzii* increased ZO-1 expression in HCT116 intestinal epithelial cells	Oral *F. prausnitzii* improved intestinal barrier function, reduced plasma LPS, and attenuated AS in ApoE^−^/^−^ mice
Wang et al. [62]	eIF6–*L. acidophilus*	Blood transcriptomic data showed higher eIF6 expression in CAD; the human gut microbiota was not assessed	—	eIF6 deficiency enriched *L. acidophilus*; *L. acidophilus* supplementation improved barrier function and attenuated AS in ApoE^−^/^−^ mice
Gan et al. [63]	Chronic apical periodontitis–oral–gut–barrier axis	—	—	Chronic apical periodontitis induced gut dysbiosis, altered bile acid metabolism, impaired the intestinal barrier, and aggravated AS in HFD-fed ApoE^−^/^−^ mice
Zhang et al. [64]	T3SS–ferroptosis	T3SS-related genes were enriched in T2DM-associated ASCVD and correlated with stenosis severity	T3SS-competent EHEC induced FTH1-related ferroptosis and barrier injury in intestinal epithelial cells	T3SS-competent *C. rodentium* aggravated intestinal injury and AS in diabetic ApoE^−^/^−^ mice
Zhu et al. [65]	3-MH–HNF1A–NPC1L1	Higher plasma 3-MH was associated with carotid plaques, cIMT, and atherogenic lipids	3-MH activated HNF1A–NPC1L1 signaling and increased cholesterol uptake in Caco-2 cells	HCD/FMT increased 3-MH; 3-MH gavage enhanced cholesterol absorption and plaque burden in ApoE^−^/^−^ mice
Nageswaran et al. [66]	ImP–PI3K/AKT–FOXO1	Higher plasma ImP was independently associated with CAD	ImP impaired endothelial migration, proliferation, and angiogenesis via PI3K/AKT inhibition and persistent FOXO1 activation	ImP impaired vascular repair and increased plaque burden and macrophage accumulation in ApoE^−^/^−^ mice
Xue et al. [67]	IPA–miR-142-5p–ABCA1	Circulating IPA was lower in CAD and inversely associated with ASCVD severity	IPA promoted macrophage cholesterol efflux via miR-142-5p inhibition and ABCA1 upregulation	IPA enhanced reverse cholesterol transport and attenuated AS in ApoE^−^/^−^ mice
Liu et al. [68]	*B. ovatus*–IAA	*B. ovatus* and IAA were reduced in ASCVD	IAA inhibited TLR4/MyD88/NF-κB signaling in M1 macrophages and promoted M2 polarization	*B. ovatus* or IAA restored barrier function, reduced vascular inflammation, and attenuated AS in ApoE^−^/^−^ mice

Abbreviations: ApoE^−^/^−^, apolipoprotein E-deficient; ASCVD, atherosclerotic cardiovascular disease; CAD, coronary artery disease; cIMT, carotid intima–media thickness; DCA, deoxycholic acid; eIF6, eukaryotic initiation factor 6; FMT, fecal microbiota transplantation; HCD, high-chicken-protein diet; HFD, high-fat diet; IAA, indole-3-acetic acid; ImP, imidazole propionate; IPA, indole-3-propionic acid; LDL, low-density lipoprotein; 3-MH, 3-methyl-L-histidine; PAGln, phenylacetylglutamine; T2DM, type 2 diabetes mellitus; T3SS, type III secretion system; TMAO, trimethylamine N-oxide. “—” indicates no corresponding evidence was reported.

**Table 3:** Microbial composition of atherosclerotic plaques.

Study	Samples	Detection Approach	Microbial Findings	Evidence Supported/Key Findings
Koren et al. [6]	Carotid atherosclerotic plaques with oral and gut samples	16S rRNA gene sequencing	*Veillonella*, *Streptococcus*, and other taxa were detected in plaques and shared with oral and/or gut samples	DNA/composition and source overlap: plaque-associated bacterial DNA showed overlap with oral and gut microbiota, supporting potential microbial connections among these sites but not proving translocation
Mitra et al. [70]	15 symptomatic carotid plaques vs. 7 stable autopsy plaques	Shotgun metagenomics + FISH	Stable plaques showed enrichment of *Porphyromonadaceae*, *Bacteroidaceae*, *Micrococcaceae* and *Streptococcaceae*; symptomatic plaques showed more *Helicobacteraceae*, *Neisseriaceae* and sulfur-related taxa; *Acidovorax* spp. and *H. pylori* were visualized by FISH	DNA/composition + spatial localization: distinct microbial signatures were observed between symptomatic and stable plaques; selected bacterial signals were spatially localized in plaque tissue
Lindskog Jonsson et al. [69]	22 symptomatic vs. 11 asymptomatic carotid plaques	16S rRNA gene sequencing	*Proteobacteria* and *Actinobacteria* predominated; modest differences were observed between symptomatic and asymptomatic plaques	DNA/composition: bacterial DNA was detected in carotid plaques, although community-level differences by symptom status were limited
Lv et al. [55]	Symptomatic carotid plaques and matched fecal samples	V3–V4 16S rRNA gene sequencing	Eleven bacterial genera enriched in the gut of patients with bacterial-DNA-positive plaques were also detected in plaques	DNA/source overlap: shared genera between fecal and plaque samples support a possible intestinal contribution, but do not establish direct translocation
Sato et al. [71]	54 carotid plaques; sequencing performed in a subset; dental plaque samples	16S rRNA gene PCR + sequencing	Core plaque genera included *Streptococcus*, *Actinomyces*, *Corynebacterium*, *Haemophilus*, and *Neisseria*; substantial overlap with oral microbiota	DNA/source overlap: substantial plaque–oral microbial overlap supports a possible oral contribution but does not demonstrate direct microbial migration or persistent colonization
Figuero et al. [72]	Vascular lesions, blood and subgingival samples from 70 patients	qPCR + nested PCR	Periodontal pathogen DNA, including *Aggregatibacter actinomycetemcomitans, P. gingivalis, T. forsythia*, and *C. rectus*, was assessed across sample types	Targeted DNA/source evidence: periodontal pathogen DNA was infrequently detected in vascular specimens; limited overlap provides only suggestive evidence for an oral source
Huang et al. [7]	33 human coronary atherosclerotic plaques	*P. gingivalis*-specific FISH	*P. gingivalis*-positive fluorescent clusters were directly localized within coronary plaques	Spatial localization: higher intraplaque *P. gingivalis* signal was associated with features of plaque vulnerability, providing direct localization rather than evidence of viability
Karhunen et al. [73]	Human coronary and surgically obtained atherosclerotic specimens	qPCR + immunohistochemistry	*Viridans* group *streptococcal* DNA and streptococcal immunoreactivity were detected within atheromatous cores, fibrous caps and plaque rupture sites	DNA + spatial localization: complementary molecular and histological evidence supports localized streptococcal signals within plaques
Esposito et al. [74]; Johnston et al. [75]	Human carotid atherosclerotic plaques	Reverse-transcription PCR	*Chlamydia pneumoniae* mRNA was detected in subsets of plaques	RNA/possible viability evidence: detection of bacterial mRNA supports possible microbial viability in selected plaques, although evidence remains limited
Lanter et al. [76]	15 human carotid artery specimens	16S rRNA gene detection + bacterial culture	*Cutibacterium* acnes (*Propionibacterium* acnes) DNA was detected in 4/15 specimens; *viable C. acnes* was among several bacterial species recovered by culture	Culture/viability evidence: recovery of viable bacteria provides stronger evidence of bacterial viability than DNA detection alone

Abbreviations: FISH, fluorescence in situ hybridization; qPCR, quantitative polymerase chain reaction; 16S rRNA, 16S ribosomal RNA.

## Data Availability

No new data were created or analyzed in this study. Data sharing is not applicable to this article.
